# Consequences of phenological shifts are determined by the number of generations per season

**DOI:** 10.1002/ecy.70454

**Published:** 2026-07-03

**Authors:** Heng‐Xing Zou, Volker H. W. Rudolf

**Affiliations:** ^1^ Program in Ecology and Evolutionary Biology, Department of BioSciences Rice University Houston Texas USA; ^2^ Institute for Global Change Biology University of Michigan Ann Arbor Michigan USA

**Keywords:** coexistence, life history, priority effects, seasonal dynamics, *Tribolium*

## Abstract

The order and timing of species' arrival in a community can often change species interactions. Termed priority effects, this phenomenon arises in communities with drastically different life histories (e.g., bacteria, insects, and amphibians), yet how their life history traits affect the consequences of priority effects in seasonal systems remains understudied. Here, we test how the length of one growing season, which can accommodate different numbers of overlapping generations, interacts with priority effects using two competing flour beetles, *Tribolium castaneum* and *Tribolium confusum*. We manipulated the sequence and timing of arrivals and collected all adults after one, two, or three generations, simulating phenological shifts in communities with different growing season lengths with less or more overlapping generations. The early‐arriving species always reached a higher population, and in general, its per capita competition towards the late arriver was stronger, indicating priority effects. However, fitting competition models to the data revealed that for both species, longer experimental durations (which included more overlapping generations) led to less dramatic changes in competitive outcomes with differences in arrival time, indicating that overlapping generations of each species provided a buffer against strong priority effects from different arrival times. These results suggest that changes in the order and timing of species arrival (e.g., due to climate change) can impact species coexistence, but such effects could be contingent on the life history, such as the number of generations of interacting species, or abiotic factors, such as the duration of a growing season.

## INTRODUCTION

The relative timing of seasonal life history events (phenology) can often change species interactions. Among competing species, this phenological difference can lead to priority effects, affecting species coexistence, and long‐term community dynamics (Fukami, 2015; Rudolf, 2019; Zou & Rudolf, 2023). Despite extensive studies, priority effects among systems with different seasonality and life histories, such as the length of the life cycle, are rarely compared and synthesized (Zou & Rudolf, 2023). These biological differences make the development of a unified theory of priority effects difficult because certain mechanisms and consequences may only apply to specific systems. Furthermore, climate and environmental changes may lead to simultaneous shifts in both phenology (Kharouba et al., 2018; Parmesan, 2006) and life histories (Møller et al., 2010; Williams et al., 2015). Yet, we lack experimental evidence on how these simultaneous changes may interact to affect community dynamics.

Many ecological communities are seasonal, meaning that species undergo certain periodic perturbations in the environment. In these seasonal communities, the number of generations is fundamental to species demography and can change plastically under different environments. Higher temperatures accelerate biological processes and can increase the number of reproductive events possible within a season across diverse taxa. For example, *Daphnia* development rate is higher in urban areas with higher temperatures, leading to a faster “pace of life” that enables more frequent reproduction (Brans & De Meester, 2018). Persistent warming increases the number of generations in a year in many European lepidopterans (Altermatt, 2010) and can also accelerate the reproduction of an invasive plant by promoting an annual rather than a biennial life cycle (Keller & Shea, 2020). Given that warming often also drives phenological shifts in a wide range of taxa, such as the emergence and arrival of species into communities or their temporal order (Parmesan, 2006), changes in the reproduction regime may occur simultaneously with changes in times of emergence for different species, which drive priority effects. These examples demonstrate why evaluating the interaction between the strengths of priority effects and the number of generations in a growing season is important under climate change.

Priority effects may occur when the phenology of two species is changed such that their relative arrival timing and order are different. In nature, priority effects can occur through two main mechanisms (Zou & Rudolf, 2023). First, the change in relative arrival times may cause a difference in size or developmental stage of the initial generation (Zou et al., 2023; Zou & Rudolf, 2023). In this mechanism, the early‐arriving species develops to a larger size or later stage before the late species arrives (colonizes or emerges from dormancy), leading to a per capita competitive advantage that alters the competitive interactions between the two species (Blackford et al., 2020; Connolly & Muko, 2003; Rasmussen et al., 2014; Shorrocks & Bingley, 1994). In experiments, these size‐ or stage‐mediated priority effects can be detected as a change in per capita competition between the two species with relative arrival times (Zou & Rudolf, 2023). These per capita coefficients can be used to predict, analytically, the long‐term effects of differences in relative arrival times on community dynamics via the calculation of two key terms: fitness difference and stabilization potential (SP) (Chesson, 2000, 2018). SP refers to the ecological differences between the two species (e.g., niche differences in resource use and habitat specialization), whereas fitness difference describes the relative performance of the two species (Barabás et al., 2018). If the per capita competition coefficients between the two species change with their relative arrival times, the calculated fitness differences and SPs will also change with relative arrival times, altering the predicted long‐term outcomes of their competition (Fragata et al., 2022; Zou & Rudolf, 2023). The stronger the size/stage‐mediated priority effects, the larger the changes in fitness differences and SPs will be, and the more likely the competitive outcomes will change.

Second, priority effects may arise when one species establishes a higher abundance by arriving earlier and maintains this advantage through positive frequency dependence (Zou et al., 2023; Zou & Rudolf, 2023). Positive frequency dependence is possible when each species limits the other more than itself, which can be predicted by a combination of negative SP and small relative fitness differences (RFDs) (Ke & Letten, 2018). When this happens, intra‐ and interspecific per capita effects predicts that two alternative stable states are possible, and which one is realized depends on the initial relative densities of both species. This mechanism does not alter per capita competitive effects, and thus changes in arrival time should not affect stabilizing potential and fitness differences. Instead, which species arrives first can affect the relative abundance of the two species, which in turn determines their competitive outcome.

Theory predicts that the number of generations in a growing season can alter the strength of priority effects among species, especially when the priority effect arises from the initial difference in sizes or stages (Zou et al., 2023). With more generations in a growing season, continuous reproduction leads to overlapping generations, and the populations of both species consist of multiple coexisting sizes/stages (Zou et al., 2023). As a season contains more overlapping generations, the size/stage distributions of the two species become less different, lowering the importance of the initial size/stage difference that determines the competition coefficients (Zou et al., 2023). As competition coefficients change less with the initial arrival times, having more generations in a season should weaken size/stage‐mediated priority effects by decreasing changes in fitness differences and SP values, eventually leading to fixed competitive outcomes regardless of the arrival times with enough generations.

However, this prediction lacks empirical evidence and is solely based on priority effects generated by size/stage difference, while in nature, they could also arise from other mechanisms. For instance, the early arriver advantage maintained through positive frequency dependence (Ke & Letten, 2018; Toju et al., 2018; Zou & Rudolf, 2023) does not require changes in competition coefficients with arrival times and therefore may not be affected by the number of generations in a season. Alternatively, differences in arrival time can allow early‐arriving species to consume smaller later arriving species (intraguild predation; Rasmussen et al., 2014), and if the predation rate is high, the late species may not survive to adulthood, leading to consistent exclusion regardless of the number of generations. Together with the different competition between sizes/stages, one or several of these mechanisms may lead to the priority effects observed between two species.

To gain insight into the interaction between the number of generations and relative arrival times in nature, we conducted an experiment with two competing flour beetles, *Tribolium castaneum* (red flour beetles) and *Tribolium confusum* (confused flour beetles). We experimentally created an arrival time gradient, then allowed the beetles to grow and reproduce for different durations to simulate different numbers of overlapping generations in a “growing season.” The arrival time gradient simulates how phenological differences in nature may lead to size/stage differences between the early and the late species, and the different numbers of overlapping generations serve as a rough proxy of potential changes in reproduction regimes due to a longer growing season under a warming climate (Altermatt, 2010) or the different numbers of generations across a latitudinal gradient from warmer climate and longer growing seasons to colder climate and shorter growing seasons (Kong et al., 2019). Although this approach does not fully capture the characteristics of phenological shifts and changes in reproduction regimes in nature, it provides a first attempt in representing these changes using a simple, tractable system. We quantified the competition coefficients for the two species at different arrival times and total durations, then analyzed the long‐term competitive outcomes predicted from fitness differences and SPs. With insight from the previous theoretical model (Zou et al., 2023), we test the hypothesis that the number of generations in a growing season weakens the strength of size/stage‐mediated priority effects.

## METHODS

### Study organisms

*T. castaneum* and *T. confusum* are common agricultural pests that feed on stored grains, but have been widely used as a model system to study population and community dynamics for a century (Edmunds et al., 2003; Park, 1948; Pointer et al., 2021). While they generally do not display seasonality and phenology in their reproduction, they compete readily in the same habitat over shared resources (Park, 1954) and display priority effects (Holditch & Smith, 2020), making them an ideal, tractable system in laboratory settings. In addition to resource competition, the two species often engage in intraguild predation and cannibalism of eggs and pupae (Alabi et al., 2008; Benoît et al., 1998).

We used a black strain of *T. castaneum* collected from Hereford, TX (J.P. Demuth), and strain PAK of *T. confusum* purchased from the United States Department of Agriculture Agricultural Research Service (Manhattan, KS). The juvenile stages (eggs, larvae, and pupae) of the two species are visually similar, but using a black strain of *T. castaneum* allows easy visual separation of the adults from *T. confusum* (red). Life history traits of the two species differ by their strains; in our experiment, *T. castaneum* has a shorter life cycle (egg to adult) than *T. confusum* (Park, 1957), but the latter produces more eggs under our experimental conditions (Appendix S1: Table S2). The life expectancy of both species ranges from 9 to 14 months, and adult females lay eggs daily throughout their life (Leslie & Park, 1949; Park, 1934), although we control for the number of overlapping generations by specific experimental treatments (see below). Beetles were reared in incubators at 32°C and 20%–40% relative humidity with constant airflow. According to pilot experiments, these environmental conditions allowed fast development while minimizing mold overgrowth. Under such conditions, we expect both species to complete their life cycle within 36 days.

### Experimental design

We collected eggs from adults reared from “sterilized eggs.” Eggs were first washed with 10% bleach solution for 30 s to 1 min, then thoroughly rinsed with sterilized deionized water and placed on filter paper for 4 h under ambient conditions to dry. We then transferred sterile eggs to a medium containing unbleached all‐purpose wheat flour (King Arthur, Norwich, VT) with 0.03% fumagillin (KBNP Inc., Anyang, South Korea) by mass. To lower the potential effects of the maternal environment (Van Allen & Rudolf, 2013), we collected eggs from newly emerged adults reared under the same environmental conditions as the main experiments. We collected eggs once per 3 days to maximize synchrony among larvae while maintaining the large number of eggs needed. We conducted all experiments by adding eggs to 7‐dram vials (29 mm diameter, 50 mm height; Bel‐Art, Warminster, PA) with 8‐g medium and perforated caps to allow for air exchange. Pilot trials showed that under the experimental densities, 8‐g medium per vial induces measurable competition. Initiating all vials with eggs better controlled the timing of species arrival and the initial density in each vial (see below).

**FIGURE 1:**
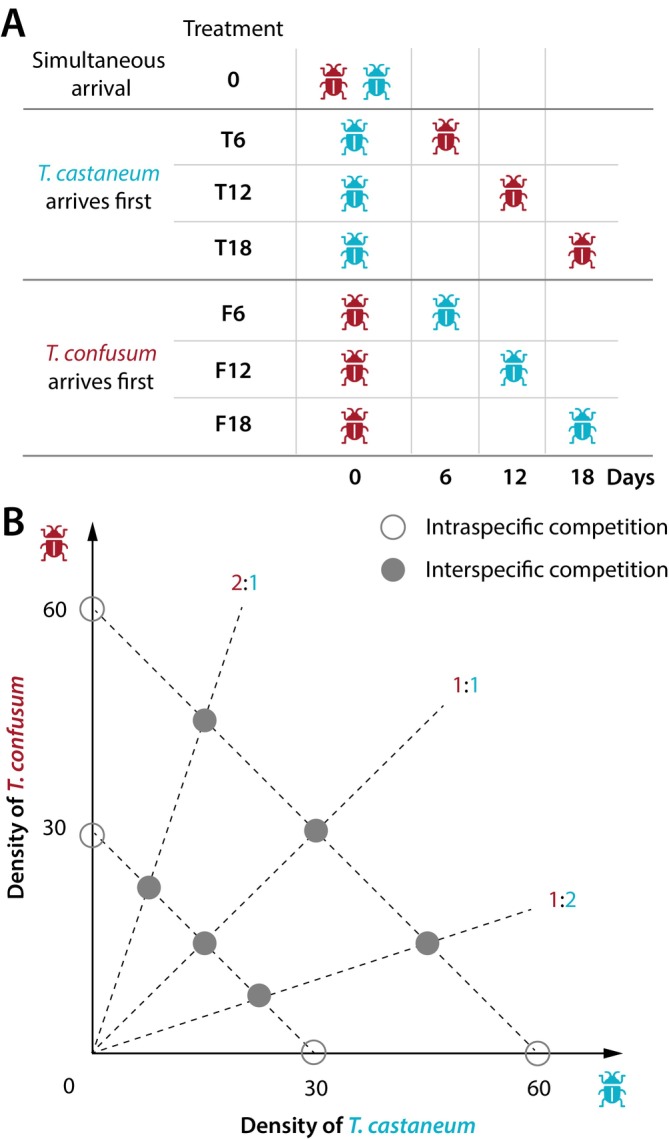
Scheme of the experiment. (A) Different arrival times (temporal treatments) of the two species, *Tribolium castaneum* and *Tribolium confusum*. (B) Response surface design with six density combinations at two total densities and three ratios, along with two single‐species treatments, to evaluate competition coefficients. Each treatment in the response surface is replicated three times. Insect icons from Microsoft 365.

We conducted a full factorial design of seven relative arrival times and three season lengths. To simulate the difference in arrival times, we added eggs of one species at Day 0 and then added eggs of the competitor either on the same day (simultaneous arrival) or 6, 12, or 18 days later, yielding seven temporal treatments (Figure 1A). We define relative arrival times as the arrival time of *T. castaneum* minus that of *T. confusum*, such that a negative value indicates the early arrival of *T. castaneum* and vice versa. We simulated different numbers of generations in a season by growing the vials for 36, 72, and 108 days, corresponding to one, two, and three generation times per season. Note that because we started all vials with eggs, the one‐generation vials were effectively a single, discrete generation, while the two‐ and three‐generation vials have overlapping generations. We determined the length of each season based on the 36 days from eggs to adults for both species under our experimental conditions. For each temporal treatment, we used a response surface design (Inouye, 2001) with two total densities (30, 60) and three density ratios (1:2, 1:1, 2:1, in the order of *T. castaneum*: *T. confusum*) to measure the per capita interspecific competition (Figure 1B), resulting in a total of 126 competition treatment scenarios (six density treatments, seven relative arrival times, and three durations). In addition, we set up single‐species vials with 30 or 60 eggs to evaluate population growth rates and intraspecific competition. Since these are single‐species treatments, they were not repeated for arrival time treatments, but they were repeated for each duration treatment. We conducted three replicates for each single‐ and two‐species treatment scenario, yielding a total of 414 experimental units (the 126 two‐species treatments plus the 12 single‐species treatments, each with three replicates). We started the experiment on May 21, 2023.

To quantify population sizes, we started counting adults at the assigned season length after adding the first species (Day 36, 72, or 108). We repeated this process every 6 days thereafter until all live individuals emerged as adults, at which time we terminated the vial. At each census, we emptied all contents in a vial to a sieve. After sieving out the medium, we transferred all individuals into a Tupperware, counted adults by visual inspection, then returned all individuals back to the vials to maintain the competitive pressure as other individuals developed, but removed eggs to terminate further reproduction. This allowed the larvae and pupae to complete their life cycle and develop into adults, at which stage we can easily identify them to species. This also ensured that arriving early would not lead to a longer time for population growth because the lengths of seasons were fixed, and eggs were removed. We did not count larvae and pupae because they look identical. All censuses were conducted by Heng‐Xing Zou.

We changed the medium of all experimental vials every 30–40 days, which roughly coincided with the first census of the adult population. If the change date did not coincide with a check date, we retained all individuals, including eggs, while also recording the number of adults. The experiment ended on October 27, 2023.

In addition to the main experiments, we conducted two complementary experiments to evaluate two key processes in our system: intraguild egg predation and fecundity. Our experimental design allowed early‐arriving larvae to consume eggs of the late‐arriving species, which is well known among *Tribolium* species (Alabi et al., 2008). We evaluated the intraguild egg predation rates by adding eggs to 6‐, 12‐, and 18‐day old larvae, simulating experimental conditions. See Appendix S1: Section S3 for detailed methods and results of both experiments.

### Statistical analysis

To quantify the competition between flour beetles, we parameterized discrete‐time Lotka–Volterra models for vials from each arrival time and experimental duration (as a proxy for the number of generations) under a Bayesian framework (Appendix S1: Section S1). Fitting Beverton‐Holt and Ricker models either led to issues with convergence (>1000 divergent transitions after warmup) or lower mean log likelihood (Appendix S1: Section S1). Key traits of flour beetles, including fecundity, are highly dependent on the natal habitat (Van Allen & Rudolf, 2013). Therefore, we cannot estimate the population growth rate of each species by measuring the fecundity of a single individual in fresh media because its natal habitat would be different from those in vials with multiple generations per season. Instead, we used information from our single‐species vials to estimate intrinsic growth rates at different experimental durations and then used this information to assist in the estimation of parameters in the two‐species model. Specifically, we treated vials with different experimental durations (numbers of generations) as a single “growing season,” meaning that within this growing season, the number of actual reproduction events is different among treatments. Therefore, instead of using all three duration treatments to fit a single intrinsic growth rate for each species, we fit single‐species Lotka–Volterra models to data obtained from single‐species vials for each duration and estimated number of generations, *G*:

(1) $$\frac{N{\left(t\right)}_{i,G}}{N{\left(0\right)}_{i,G}}=\lambda_{i,G}\left(1-\alpha_{\mathrm{ii},G}N{\left(0\right)}_{i,G}\right),$$

where $N{\left(t\right)}_{i,G}$ is the final adult density of species i with G generations, $N{\left(0\right)}_{i,G}$ is the initial egg density, $\lambda_{i,G}$ is the “effective” growth rate of species i in a growing season consisting of G generations, and $\alpha_{\mathrm{ii},G}$ is the per capita intraspecific competition coefficient. In this step, we used flat priors for $\lambda_{i,G}$ and $\alpha_{\mathrm{ii},G}$ but confined the latter from 0 to 1. We then used the fitted effective growth rates to inform the model fitting of two‐species Lotka–Volterra models as priors:

(2) $$\frac{N{\left(t\right)}_{i,G}}{N{\left(0\right)}_{i,G}}=\lambda_{i,G}\left(1-\alpha_{\mathrm{ii},G}N{\left(0\right)}_{i,G}-\alpha_{\mathrm{ij},G}N{\left(0\right)}_{j,G}\right),$$

where $\alpha_{\mathrm{ij},G}$ is the interspecific competition coefficient from species j to species i. For the prior, we constructed a normal distribution with the mean as the posterior estimates of each $\lambda_{i,G}$ and the standard deviation as 10% of the mean $\lambda_{i,G}$ (see Appendix S1: Figure S6 for fitted intrinsic growth rates). Using these more constrained values did not change the qualitative results but greatly helped model convergence. We repeated the above process, fixing the priors of $\lambda_{i,G}$ for all vials with G generations, but used flat priors for the competition coefficients $\alpha_{\mathrm{ii},G}$ and $\alpha_{\mathrm{ij},G}$ from 0 to 1 to allow for their variations at different relative arrival times. This approach assumes that the effective growth rate changes with the experimental duration (number of generations) but not relative arrival times. This is the most likely biological scenario because the number of generations is naturally linked with the reproductive outcome, while any effect of relative arrival times on the maternal age and the environment will be reflected by competition coefficients.

Finally, to characterize the long‐term competitive outcomes for each combination of arrival time and season length treatments, we calculated the SP and RFDs from the four competition coefficients from each of the treatment scenarios (density treatments, relative arrival time treatments, and duration treatments; Godwin et al., 2020; Ke & Letten, 2018). We randomly subsampled 1000 posterior estimates of each competition coefficient from the model fitting results. For each subsample, we calculated SP and RFD as follows:

(3) $$\mathrm{SP}=1-\sqrt{\frac{\alpha_{\mathrm{ij}}\alpha_{\mathrm{ji}}}{\alpha_{\mathrm{ii}}\alpha_{\mathrm{jj}}}},$$

(4) $$\mathrm{RFD}=\sqrt{\frac{\alpha_{\mathrm{ij}}\alpha_{\mathrm{ii}}}{\alpha_{\mathrm{jj}}\alpha_{\mathrm{ji}}}},$$

We report the mean and standard deviation of these values. Coexistence requires that the two species cannot be too ecologically similar (positive SP), and one should not perform much better than the other (small RFD), or specifically: $1-\mathrm{SP}<\mathrm{RFD}<\frac{1}{1-\mathrm{SP}}$. If this inequality is reversed, that is, $1-\mathrm{SP}>\mathrm{RFD}>\frac{1}{1-\mathrm{SP}}$, the two species display positive frequency dependence (Ke & Letten, 2018; Zou & Rudolf, 2023), that is, the species with a higher initial abundance wins.

We conducted all analyses in R 4.2.1 (R Core Team, 2024) and fitted the Bayesian model in Stan using the package rstan (Stan Development Team, 2024). We fitted all models with a total of 100,000 iterations, using the first 50,000 as burn‐in. All models converged well (all $\hat{r}$ values <1.0001; visual examination of all trace plots indicated that the chains were well mixed). Data and code are available in Zou (2026) at https://doi.org/10.6084/m9.figshare.32118556.v2.

## RESULTS

### Priority effects and competition coefficients

**FIGURE 2:**
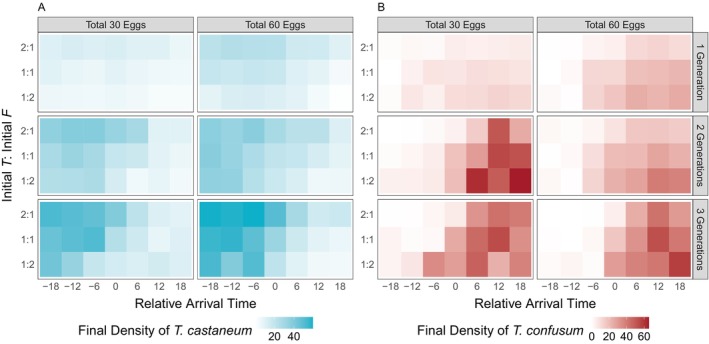
Final adult populations in vials with two species (panel (A). *Tribolium. castaneum*, or T; panel (B). *Tribolium confusum*, or F), by total densities, the ratio of initial egg densities, the number of generations, and relative arrival times. On the *x*‐axis, a negative relative arrival time indicates the early arrival of *T. castaneum*, a positive relative arrival time indicates the early arrival of *T. confusum*, and a 0 indicates simultaneous arrival. The *y*‐axis represents different ratios of the initial egg densities, in the order of *T. castaneum* (T): *T. confusum* (F). The adult population is averaged across the three replicates. A darker shade indicates a higher adult population.

In general, the earlier a species arrived, the larger its final population (i.e., the number of adults at the end of the one‐, two‐, or three‐generation experimental periods), indicating strong priority effects (Figure 2; Appendix S1: Figures S3–S5; see also Appendix S1: Section S2). Without the competing species, *T. confusum* reached higher densities than *T. castaneum*, suggesting overall higher fecundity (Appendix S1: Table S1), but *T. castaneum* appeared competitively dominant because it could completely exclude *T. confusum* when arriving early (Appendix S1: Figures S3–S5). See Appendix S1: Section S2 for further analyses on the final adult population.

**FIGURE 3:**
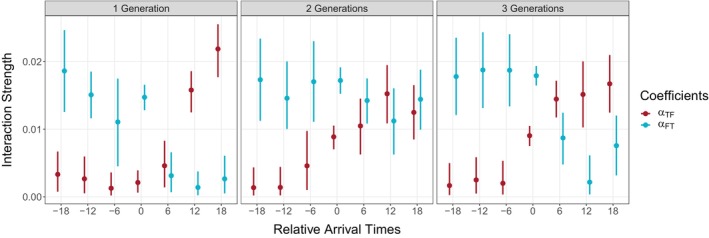
Fitted interspecific competition coefficients in a discrete‐time Lotka–Volterra model. $\alpha_{\mathrm{TF}}$ indicates the effect of *Tribolium confusum* to *Tribolium castaneum*, and vice versa. A negative relative arrival time indicates the early arrival of *T. castaneum*, a positive relative arrival time indicates the early arrival of *T. confusum*, and a 0 indicates simultaneous arrival. Points show the median (50%), and line ranges show 10% and 90% of the posterior distribution.

Fitted interspecific per capita competition coefficients further illustrated the presence of priority effects. The earlier a species arrived, the stronger its per capita competitive effect towards the late arriver (Figure 3), and *T. confusum* needed to arrive earlier to achieve equal interspecific competition between *T. castaneum*. This suggests that *T. castaneum* was competitively dominant in general. This priority effect was consistent with the differences in egg predation observed in our predation trials: The larvae of *T. castaneum* had a higher predation rate on the eggs of *T. confusum*, with larger larvae consuming more eggs (Appendix S1: Figure S7). However, the duration of the experiment (number of generations) did not have a consistent effect on the strength of priority effects (Figure 3): Although the two competition coefficients were more similar when *T. confusum* arrived earlier with two generations per season, this similarity was absent with three generations per season.

### Stabilization potential and fitness differences

**FIGURE 4:**
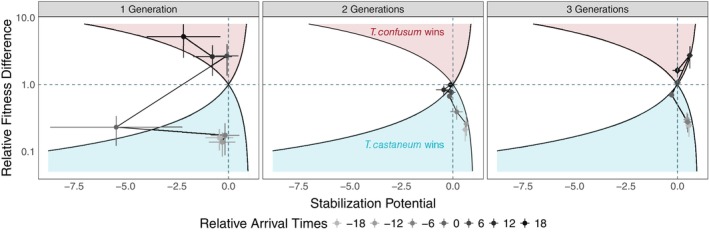
Stabilization potentials and relative fitness differences calculated from 1000 randomly sampled posterior estimates of intra‐ and interspecific competition coefficients. A negative relative arrival time indicates the early arrival of *Tribolium castaneum* (lighter shaded points), a positive relative arrival time indicates the early arrival of *Tribolium confusum* (darker shaded points), and a 0 indicates simultaneous arrival. Points show values calculated by the median, and line ranges show values calculated by 10% and 90% of the posterior distribution of the competition coefficients. Points are associated with the relative arrival times that they represent. The blue shaded region below the solid curves indicates *T. castaneum* wins, the red shaded region above the solid curves indicates *T. confusum* wins. The region to the left of both curves indicates frequency‐dependent priority effects. The region to the right indicates coexistence.

Calculated SP and RFDs showed much larger variations across different arrival times when the experiment ran for one generation as compared to two or three generations (Figure 4). With one generation, mean SPs were always negative, indicating a system with a strong tendency towards positive frequency dependence (Ke & Letten, 2018). Combined with strong RFDs, we observed a strong tendency towards exclusion when either species arrived early. At simultaneous arrival, the two species tended to display positive frequency dependence. With more generations, both SP and fitness differences between the species were much smaller but still generally predicted exclusion when either species arrived early. With simultaneous arrival and experimental durations of two or three generations, *T. castaneum* tended to exclude *T. confusum*, matching our expectation that the former was competitively superior. With two generations, when *T. confusum* arrived early by 12 and 18 days, the overall SP and fitness differences predicted positive frequency dependence, a result different from durations of both one and three generations. This likely arose from the particularly high population of *T. confusum* when it arrived early and had a numeric advantage (Appendix S1: Figures S3–S5), which affected the fitted competition coefficients and, subsequently, SPs and fitness differences. With three generations, the early arrival of either species led to more positive SP values, suggesting a stabilizing effect of arriving at different times. However, the two species were still predicted to exclude each other due to the high fitness differences.

## DISCUSSION

As we strive to predict the consequences of phenological shifts across systems, understanding the roles of seasonality in modulating these effects among systems with different reproduction regimes is fundamental. We explored how experimental duration, which determined the length and subsequently the number of generations in a “growing season,” shapes the competitive dynamics between two flour beetles as they arrive at different times. With different relative arrival times, we found strong changes in interspecific competition via changes in SPs and fitness differences, indicating size/stage‐mediated priority effects. Furthermore, running the experiment for more generations led to increasingly positive SPs and smaller RFDs between the two species, indicating a decrease in such priority effects. These results show the nuanced relationship between the consequences of relative arrival times and the number of overlapping generations allowed in growing seasons of different lengths.

### Separating different mechanisms of priority effects

Increasing experimental evidence suggests that priority effects can arise from changes in species interactions with their arrival times, often due to changes that affect these biotic interactions, such as sizes or stages (Blackford et al., 2020; Fragata et al., 2022; Rudolf, 2018; Shorrocks & Bingley, 1994). We observed changes in the estimated competition coefficients with the relative arrival times between the two flour beetles, indicating strong size/stage‐mediated priority effects (Zou & Rudolf, 2023), acknowledging that this observation applies to changes in competition coefficients fit to phenomenological Lotka–Volterra models that lack biological mechanisms. Although we did not directly test for specific traits that might have been changed by arriving early or late among the flour beetles, we identified several potential biological mechanisms. All stages of flour beetles compete readily for resources, and the later stages (larger larvae and adults) should consume resources at higher rates than smaller larvae (Maino & Kearney, 2015). Because the earlier arriving species had more time to increase in relative size/stage, it gained a competitive advantage, leading to priority effects (Zou et al., 2023). In addition to resource competition, larvae of the early arriver may consume eggs of the late arriver after the experimental egg addition, further enhancing priority effects (Alabi et al., 2008). Indeed, we found that larger larvae of *T. castaneum* consumed more eggs of late‐arriving *T. confusum* than larvae of *T. confusum* consumed of late‐arriving *T. castaneum*; moreover, egg consumption by *T. confusum* did not increase at larger larval sizes. This asymmetric egg consumption led to an even stronger early arriver advantage for *T. castaneum* through intraguild predation. When combined with other effects such as resource competition mediated by larval sizes, *T. castaneum* could drive *T. confusum* extinct in just one generation. Together, these mechanisms strengthened priority effects between the two species, as indicated by both fitted competition coefficients and calculated SP and fitness differences across relative arrival times (Figures 3 and 4). Although in both species larvae and adults may cannibalize eggs and pupae (Benoît et al., 1998), the consequences of cannibalism on community dynamics may not be as evident in the time frame considered by our experiments (1–3 generations) compared to intraguild predation, which directly affected the final population of the other species.

In addition to these mechanisms, priority effects can also arise from a higher abundance of the early‐arriving species, which can be further maintained by positive frequency dependence (i.e., frequency‐dependent priority effects; Ke & Letten, 2018; Toju et al., 2018; Zou & Rudolf, 2023). Importantly, this mechanism of priority effects is not mutually exclusive with the size/stage‐mediated priority effects. Positive frequency dependence happens when the two species limit each other more than themselves and is characterized by a negative SP and a small RFD (Ke & Letten, 2018). Because SP and RFD are calculated from competition coefficients, changes in size/stage‐mediated priority effects that change the competition coefficients with different arrival times can move values of SP and RFDs into the range that gives rise to positive frequency dependence (Zou & Rudolf, 2023). Indeed, previous experiments on *Tribolium* beetles found that the species with higher initial abundance usually wins (Park, 1957). In our experiments, the initial frequency of the two flour beetles affected the final adult population sizes, indicating the presence of frequency dependence. SPs and RFDs further suggested that positive frequency dependence was likely to occur when the two species arrived simultaneously and the experiment was run for just one generation per season or when *T. confusum* arrived very early when the experiment was run for two generations. These results indicate that in the flour beetle system, priority effects can be generated by both the difference in size/stages and the initial abundance, corresponding to previous theoretical and empirical results that the two different mechanisms (trait‐dependent and frequency‐dependent priority effects; see Zou & Rudolf, 2023 for definitions of the terms) can concurrently shape community dynamics (Fragata et al., 2022; Zou et al., 2023).

### Nuanced effects of the number of generations on phenological differences

Previous models predicted that more overlapping generations within a species in a season should reduce the difference in stage distributions of the two species and thus lower the strength of size/stage‐mediated priority effects (Zou et al., 2023). Yet, our experiment found a more nuanced response: Despite the general *presence* of priority effects observed from per capita interaction strengths for all experimental durations, the weakened priority effects are only visible from calculated SP and RFDs. To better understand the discrepancies between the theoretical prediction and the experimental outcomes, we need to identify the possible biological mechanisms that caused the signs and strengths of observed SP and RFDs across different numbers of generations.

A positive SP indicates that species limit themselves more than each other (e.g., via resource partitioning), which should promote stable coexistence. A negative SP indicates that species limit each other more than themselves, which promotes alternative stable states of competitive exclusion (see Ke & Letten, 2018 for a semantic discussion on the terminology). On the other hand, a smaller fitness difference “equalizes” the competitive ability between two species, promoting coexistence when SP is positive (Chesson, 2000, 2018). In our experiment, the strongly negative SP when the experiment lasted one generation indicated positive frequency dependence for both species, especially when they arrived at the same time (Figure 4). This lack of coexistence is expected because the two species are known to strongly compete through either resource competition (Park, 1948), interference (e.g., “conditioning” of medium via changes in structure and chemicals; Bullock et al., 2020; Ghent, 1963), or intraguild predation (Alabi et al., 2008). However, SP s became less negative and even switched to positive values with more generations, suggesting that arriving at different times promoted some form of niche differentiation between the two species. One possible source of such niche differentiation is that a large initial difference in arrival times may exempt the larvae of late‐arriving species from some resource competition when the early‐arriving species pupate and do not forage. However, this difference in size/stage distribution should decline with more overlapping generations, yet we observed increasing SP instead, suggesting other unaccounted mechanisms.

Several biological characteristics could lead to fitness differences between the two species, and their changes over experimental durations (numbers of generations). *T. castaneum* develops faster (Park, 1948, 1954) and, in our experiment, consumed more eggs of *T. confusum*. *T. confusum* always reached higher population in single‐species vials due to its higher fecundity in our experiment and higher tolerance to unfavorable environmental conditions (high population density, low availability of resources, or flour previously “conditioned” by other beetles; Riddle, 1976; Riddle et al., 1986). With only one generation, the fast development and high egg predation rate made *T. castaneum* competitively dominant, sometimes leading to the extinction of *T. confusum*. This explains why we observed strong fitness differences between the two species with only one generation, even when the two species arrived at the same time. However, if enough *T. confusum* survived to adulthood after the first generation (more likely when *T. confusum* is initially more abundant), their higher fecundity could buffer against the high predation rate of their eggs by *T. castaneum*, leading to apparent coexistence in the experimental vials. Therefore, more generations led to smaller fitness differences and weaker competitive asymmetry. This buffering could only happen in vials with more than one generation and could potentially explain why patterns in SP and fitness differences only changed qualitatively from one to two generations per season. This buffering could even increase with more generations because longer experimental durations may allow for additional reproduction events that further increase the final population of *T. confusum*. This could potentially explain why *T. confusum* was unable to gain an early arriver advantage by having similar competition coefficients in two‐generation vials but was able to regain the early arriver advantage in three‐generation vials.

### Implications and future directions

Despite extensive experimental and theoretical explorations, few studies consider the consequences of priority effects in the context of repeated disturbance followed by reassembly of communities (Zou & Rudolf, 2023). This periodic reassembly is widespread in seasonal ecosystems and may lead to compositional cycles or alternative transient states, with long‐term dynamics that depend on the strengths of species interactions (Song, 2025; Song et al., 2021; Spaak & Schreiber, 2023). Therefore, exploring the effects of key processes of seasonal communities, such as dormancy (Wisnoski et al., 2019), stochasticity (Stump & Vasseur, 2023), and reproduction regimes (Knell & Thackeray, 2016), on species interactions is important to predict long‐term dynamics of ecological communities. Although we did not examine periodic reassembly, our experiment focuses on the reproduction regime and length of a growing season and provides a first empirical test of recent theoretical exploration of the interaction between the number of generations per season and the strengths of size/stage‐mediated priority effects (Zou et al., 2023). Instead of the increasing dominance of positive frequency dependence predicted by the theoretical model, we found sustained but weakened size/stage‐mediated priority effects in our experiments. This deviation might arise from the relatively shorter duration of our experiment, but more importantly, it shows that even in a controlled system, empirical systems may contain biotic processes that are unaccounted for in theoretical models. Given the prevalence and importance of seasonal communities in nature, our results further highlight the importance of testing theoretical predictions of periodic reassembly by experiments.

Life history traits, such as the number of reproductive events per growing season, can be highly plastic in response to environmental changes (Altermatt, 2010; Brans & De Meester, 2018; Keller & Shea, 2020). Such plasticity can reflect either a warming climate over time or variations in climate across latitudinal gradients (Kong et al., 2019). Studying the community‐level consequences of potentially simultaneous shifts in both phenology and life history traits is therefore fundamental to understanding nature's responses to climate change. We found weakened size/stage‐mediated priority effects with longer seasons that allow for more overlapping generations. These results suggest that the consequences of phenological shifts, as manifested in the form of priority effects, may change in systems with different numbers of generations in a growing season, such as a warming habitat, or similar communities across a climatic gradient (e.g., latitudinal gradient from south to north). Our results join a handful of theoretical models (Knell & Thackeray, 2016; Zou et al., 2023) in presenting evidence of the interacting effects between phenological shifts and changing life history traits. However, our experiment only simulated the shift in the reproduction regime by manually imposing the length of a growing season. Further experiments that can induce such changes in life history by directly manipulating the environment are needed to better characterize the interaction between life history change and priority effects. Together, our results can assist the development of a unified theory of priority effects across different communities and the evaluation of priority effects under the synergistic shifts of phenology and life history under climate change.

## AUTHOR CONTRIBUTIONS

Heng‐Xing Zou conceived the idea and developed the methods with assistance from Volker H. W. Rudolf. Heng‐Xing Zou conducted the experiment, performed analyses with assistance from Volker H. W. Rudolf, and wrote the first draft. Both authors contributed to revisions.

## CONFLICT OF INTEREST STATEMENT

The authors declare no conflicts of interest.

## Supporting information


Appendix S1:

## Data Availability

Data and code (Zou, 2026) are available in Figshare at https://doi.org/10.6084/m9.figshare.32118556.v2.
