# Supplementary material for: Consequences of phenological shifts are determined by the number of generations per season
Source: Ecology. 2026 Jul 3;107(7):e70454. doi: 10.1002/ecy.70454 (PMC13371149; doi:10.1002/ecy.70454)
Supplement: Supplementary file 1 — Appendix S1: [file ECY-107-e70454-s001.pdf]

Appendix S1

Consequences of phenological shifts are determined by the number of generations per season

Heng-Xing Zou, Volker H.W. Rudolf

Ecology

Section S1: Bayesian Model Fitting

We fit the final count of the adults of species *i* with number of generations *G* as:

$$C(t)_{i,G} \sim Normal(N_{i,G}, \sigma) \qquad \text{(S1).}$$

We used a normal distribution because within each treatment of the number of generations, fitting the model with a Poisson or negative binomial distribution led to convergence issues (>1000 divergent transitions after the warmup). We then fitted two alternative models, Beverton-Holt (Eqn. S2) and Ricker (Eqn. S3), to one dataset from our experiments (single-species vials of *T. castaneum*, one generation, *n*=6):

$$\frac{N(t)_{i,G}}{N(0)_{i,G}} = \frac{\lambda_{i,G}}{1+\alpha_{ii,G}N(0)_{i,G}} \qquad \text{(S2);}$$

$$\frac{N(t)_{i,G}}{N(0)_{i,G}} = exp\left(\lambda_{i,G}\left(1 - \alpha_{ii,G}N(0)_{i,G}\right)\right) \qquad \text{(S3).}$$

We then calculated the average log likelihood of the posterior. Both Beverton-Holt and Ricker models had convergence issues and lower log likelihood (-4.012 and -4.502) than a discrete Lotka-Volterra model (-2.926). We therefore chose the Lotka-Volterra model (Eqn. 1 in the main text) to fit all the datasets.

To further evaluate the goodness of model fitting, we compared the fitted growth rate, $\frac{\widehat{N(t)_{i,G}}}{N(0)_{i,G}} = \widehat{\lambda_{i,G}}\left(1 - \widehat{\alpha_{ii,G}}N(0)_{i,G}\right)$, where parameters with the hat are fitted from the model, to the actual growth rate calculated from experimental data. Fitted growth rates generally match well with the actual data (Figures S1, S2), except when one species is extremely favored/unfavored. For instance, when *T. castaneum* arrives early its actual growth rate can greatly exceed the fitted growth rate, at which the actual growth rate of *T. confusum* is lower than fitted. This is because the advantage of *T. castaneum* was so large that population of *T. confusum* was close to 0.

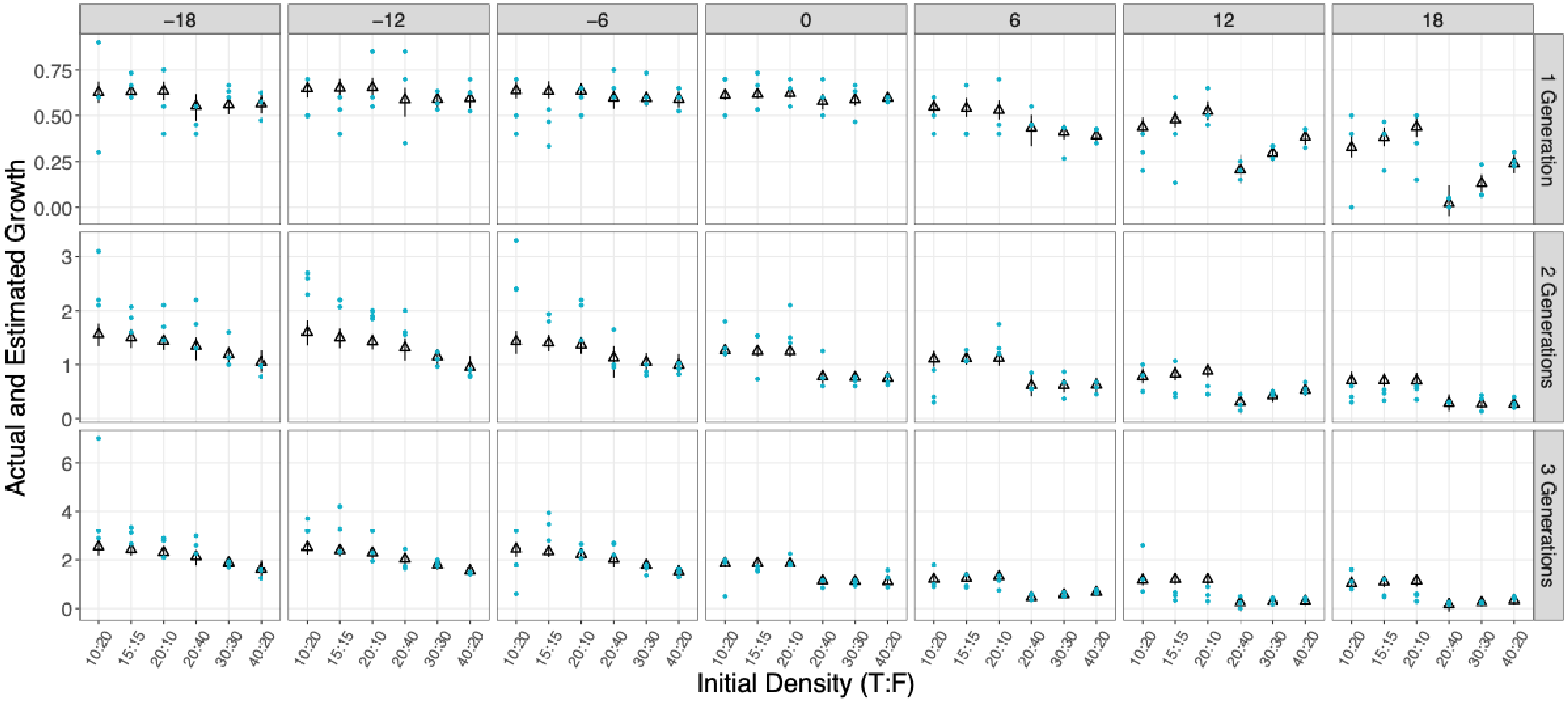

Figure S1. Actual and fitted growth rates (N(t)/N(0)) of *T. castaneum*. Rows represent number of generations and columns represent relative arrival times. Shaded intervals show growth rates calculated from 1000 randomly sampled sets of parameters, and darker to lighter shades show the 50%, 80%, and 95% rates around the median. Blue points show actual growth rates in the experiments. Initial egg densities (*T. castaneum* to *T. confusum*) are shown on the x axis.

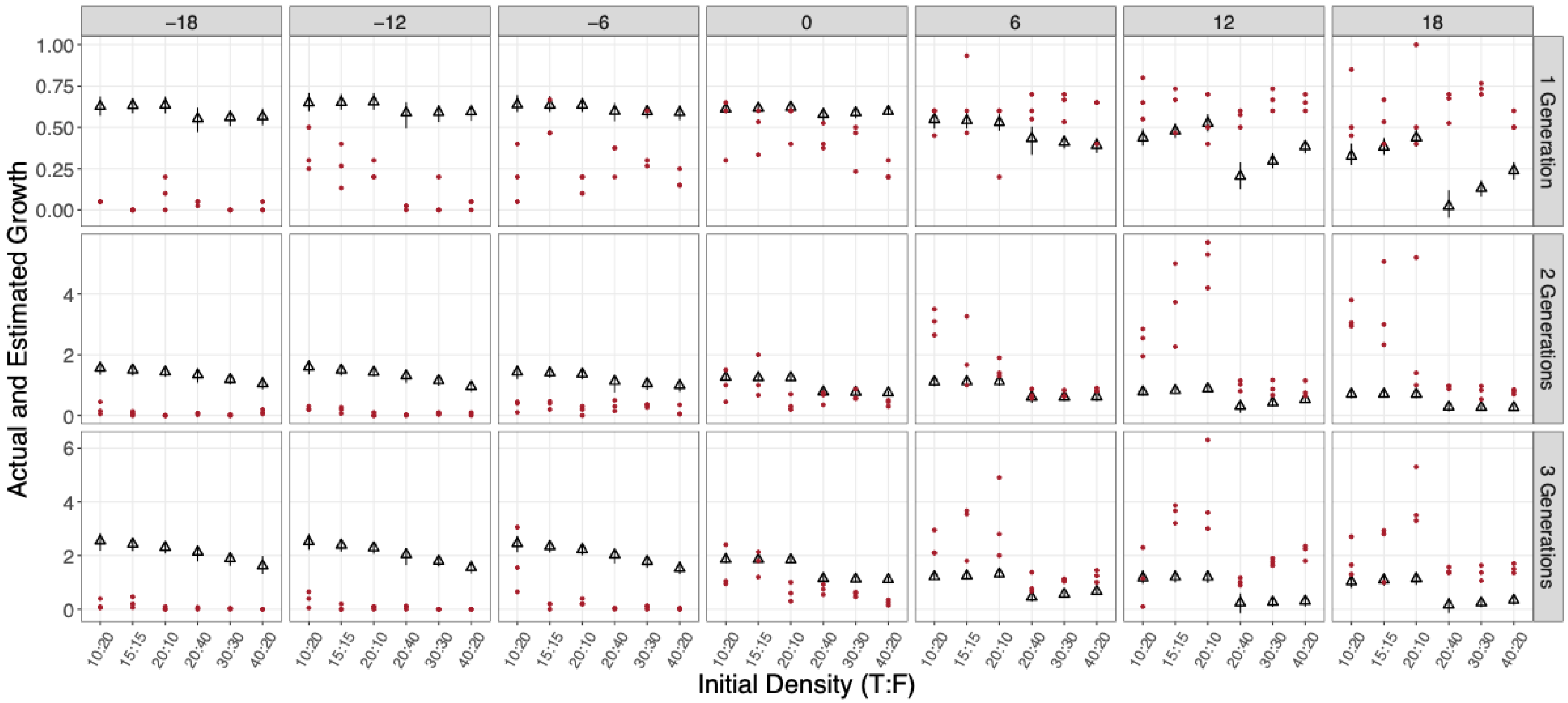

Figure S2. Actual and fitted growth rates (N(t)/N(0)) of *T. confusum*. Rows represent number of generations and columns represent relative arrival times. Shaded intervals show growth rates calculated from 1000 randomly sampled sets of parameters, and darker to lighter shades show the 50%, 80%, and 95% rates around the median. Red points show actual growth rates in the experiments. Initial egg densities (*T. castaneum* to *T. confusum*) are shown on the x axis.

Section S2: Final Adult Population

Visually inspecting the final adult populations (Figure 2, Table S1, Figures S3-S5), both species gained higher populations when they arrive earlier regardless of the treatments, indicating a strong priority effect. Note that we analyze priority effects more quantitatively by examining the competition coefficients and predicted long-term competition outcomes (see Main Text), and the analysis on final adult populations presented below only serve as a statistical check to the priority effects that are visually apparent from the final adult populations.

We first fitted linear models for each species, with the final adult population of species i (N_i) as the response variable and relative arrival time (Δt), the number of generations (G), and the ratio of initial egg densities (R) as fixed predictor variables:

$$N_i \sim \Delta t + G + R \quad \text{(S4).}$$

Alternatively, we included the interaction of all fixed predictor variables:

$$N_i \sim \Delta t + G + R + \Delta t \times G + \Delta t \times R + G \times R \quad \text{(S5).}$$

In both models, the ratio of initial egg densities was fitted as a categorical variable. The model without interactive effects (Eqn. S4) served as a “sanity check” on whether all treatments affect the final adult population. Because we expected priority effects to change across treatments, we expected many interactions terms in Eqn. S5 would be statistically significant. Adding random effects of replicate vials did not improve model fitting (results not shown).

Fitting Model 1, we found that the final adult population of both species decreased significantly when it arrived later, indicating strong priority effects (*T. castaneum*: estimated slope = -0.78, 95% CI = [-0.85, -0.70]; *T. confusum*: estimated slope = 0.99, 95% CI = [0.89, 1.08]; slopes differ in sign because negative relative arrival times indicate early arrival of *T. castaneum*, and positive relative arrival times indicate early arrival of *T. confusum*). More

generations in a season led to significantly higher total populations of both species (*T. castaneum*: estimated slope = 9.77, 95% CI = [8.67, 10.87]; *T. confusum*: estimated slope = 6.28, 95% CI = [4.85, 7.70]). For both species, an initial numeric advantage led to up to 10-fold increases of final population: increasing the initial relative abundance of *T. castaneum* from 1:2 to 1:1 and 2:1 increased its final population by 4.94 times (95% CI = [2.74, 7.15]) and by 10.57 times (95% CI = [8.37, 12.78]) and decreased the final population of *T. confusum* by 3.89 times (95% CI = [-6.74, -1.04]), and by 9.86 times (95% CI = [-12.71, -7.01]), respectively (see Table S2 for full statistics). Fitting Model 2, we found that the slopes for relative arrival times for both species are positive, which was different from results of Model 1 and our expectation that earlier arrival should lead to higher final populations. This was likely due to the inclusion of the interactive effects, which are sometime strong and statistically significant (see Table S3 for full statistics). From our observation, the number of generations might have contributed more to the final adult populations, which was also reflected by many significant interactions between number of generations and relative arrival times (Figure S3).

To confirm the effects of relative arrival times on final adult populations, we further fit linear models separately for each species and number of generations, without the interaction term between relative arrival times and ratio of initial egg densities:

$$N_{i,G} \sim \Delta t + R \qquad \text{(S6)},$$

where $N_{i,G}$ is the final population for species i with number of generations G. Results show that for all species and number of generations, earlier arrival led to larger adult populations, agreeing with our expectation and visual patterns of the data (see Table S4 for full statistics).

Table S1. Final densities of single-species vials.

Species	Number of Generations	Initial Population	Average Final Population	Standard Error (n=3)
T. castaneum	1	30	15.67	0.88
		60	38.33	1.20
	2	30	34.67	0.88
		60	43.33	0.88
	3	30	56.00	3.21
		60	62.00	3.21
T. confusum	1	30	20.00	2.00
		60	38.00	1.15
	2	30	71.00	6.11
		60	57.33	4.05
	3	30	68.67	5.55
		60	69.00	1.73

Table S2. Summary statistics of the linear effects model without interaction terms (Eqn. S4) for the final adult population of each species. Ratio represents the ratio of initial egg densities (1:1, 1:2, 2:1); it was fitted as a categorical variable and is always in the order of *T. castaneum* : *T. confusum*.

**Species**	**Variable**	**Estimate (95% CI)**	**Standard Error**	**Degree of Freedom**	**t Value**	***p***
T. castaneum	(Intercept)	-3.55 (-6.25, -0.85)	1.37	373	-2.58	0.010
	Number of Generations	9.77 (8.67, 10.87)	0.56	373	17.43	**<0.0001**
	Relative Arrival Times	-0.78 (-0.85, -0.70)	0.038	373	-20.42	**<0.0001**
	Ratio 1 (1:1)	4.94 (2.74, 7.15)	1.12	373	4.41	**<0.0001**
	Ratio 2 (2:1)	10.57 (8.37, 12.78)	1.12	373	9.43	**<0.0001**
	N = 378; groups: Replication, 3; multiple R-squared: 0.68; adjusted R-squared: 0.68					
T. confusum	(Intercept)	8.28 (4.79, 11.77)	1.78	373	4.66	**<0.0001**
	Number of Generations	6.28 (4.85, 7.70)	0.73	373	8.66	**<0.0001**
	Relative Arrival Times	0.99 (0.89, 1.08)	0.049	373	20.02	**<0.0001**
	Ratio 1 (1:1)	-3.89 (-6.74, -1.03)	1.45	373	-2.68	**0.0077**
	Ratio 2 (2:1)	-9.86 (-12.71, -7.00)	1.45	373	-6.80	**<0.0001**
	N = 378; groups: Replication, 3; multiple R-squared: 0.58; adjusted R-squared: 0.58					

Table S3. Summary statistics of the linear effects model with interaction terms (Eqn. S5) for the final adult population of each species. Ratio represents the ratio of initial egg densities (1:1, 1:2, 2:1); it was fitted as a categorical variable and is always in the order of *T. castaneum* : *T. confusum*.

**Species**	**Variable**	**Estimate (95% CI)**	**Standard Error**	**Degree of Freedom**	**t Value**	***p***
T. castaneum	(Intercept)	-0.84 (-4.08, 2.40)	1.65	368	-0.51	0.61
	Number of Generations	8.42 (6.92, 9.92)	0.76	368	11.04	**<0.0001**
	Relative Arrival Times	0.39 (0.21, 0.57)	0.090	368	4.34	**<0.0001**
	Ratio 1 (1:1)	1.83 (-2.75, 6.40)	2.33	368	0.78	0.43
	Ratio 2 (2:1)	5.57 (0.99, 10 15)	2.33	368	2.39	**0.017**
	Num Gen : Rel Arriv Times	-0.56 (-0.63, -0.48)	0.037	368	-15.15	**<0.0001**
	Num Gen : Ratio 1 (1:1)	1.56 (-0.56, 3.68)	1.08	368	1.45	0.15
	Num Gen : Ratio 2 (2:1)	2.50 (0.38, 4.62)	1.08	368	2.32	**0.021**
	Rel Arriv Times : Ratio 1 (1:1)	-0.099 (-0.24, 0.46)	0.073	368	-1.34	0.18
	Rel Arriv Times : Ratio 2 (2:1)	-0.074 (-0.22, 0.070)	0.073	368	-1.01	0.31
	N = 378; groups: Replication, 3; multiple R-squared: 0.81; adjusted R-squared: 0.80					
T. confusum	(Intercept)	7.12 (2.27, 11.97)	2.47	368	2.89	**0.0041**
	Number of Generations	6.86 (4.61, 9.10)	1.14	368	6.01	**<0.0001**
	Relative Arrival Times	0.19 (-0.078, 0.45)	0.13	368	1.39	0.17
	Ratio 1 (1:1)	-2.87 (-9.72, 3.99)	3.49	368	-0.82	0.41
	Ratio 2 (2:1)	-7.40 (-14.26, -0.55)	3.49	368	-2.12	**0.034**
	Num Gen : Rel Arriv Times	0.47 (0.36, 0.58)	0.055	368	8.54	**<0.0001**
	Num Gen : Ratio 1 (1:1)	-0.51 (-3.69, 2.66)	1.61	368	-0.32	0.75
	Num Gen : Ratio 2 (2:1)	-1.23 (-4.40, 1.95)	1.61	368	-0.76	0.45
	Rel Arriv Times : Ratio 1 (1:1)	-0.080 (-0.30, 0.14)	0.11	368	-0.73	0.46
	Rel Arriv Times : Ratio 2 (2:1)	-0.33 (-0.55, -0.12)	0.11	368	-3.02	**0.0027**
	N = 378; groups: Replication, 3; multiple R-squared: 0.66; adjusted R-squared: 0.66					

Table S4. Summary statistics of the linear effects model for the final adult population of each species and at each generation length (Eqn. S6). Ratio represents the ratio of initial egg densities (1:1, 1:2, 2:1); it was fitted as a categorical variable and is always in the order of *T. castaneum* : *T. confusum*.

**Species**	**Number of Generations**	**Variable**	**Estimate (95% CI)**	**Standard Error**	**DF**	**t Value**	***p***
T. castaneum	1	(Intercept)	6.86 (5.45, 8.26)	0.71	122	9.65	**<0.0001**
		Relative Arrival Times	-0.23 (-0.30, -0.16)	0.034	122	-6.70	**<0.0001**
		Ratio 1 (1:1)	3.83 (1.84, 5.82)	1.01	122	3.82	**0.00022**
		Ratio 2 (2:1)	8.33 (6.34, 10.32)	1.01	122	8.29	**<0.0001**
		N = 126; groups: Replication, 3; multiple R-squared: 0.48; adjusted R-squared: 0.47					
	2	(Intercept)	17.43 (15.72, 19.14)	0.86	122	20.20	**<0.0001**
		Relative Arrival Times	-0.76 (-0.85, -0.69)	0.042	122	-18.48	**<0.0001**
		Ratio 1 (1:1)	4.05 (1.63, 6.46)	1.22	122	3.32	**0.0012**
		Ratio 2 (2:1)	10.05 (7.63, 12.46)	1.22	122	8.23	**<0.0001**
		N = 126; groups: Replication, 3; multiple R-squared: 0.77; adjusted R-squared: 0.77					
	3	(Intercept)	23.69 (20.73, 26.65)	1.49	122	15.85	**<0.0001**
		Relative Arrival Times	-1.34 (-1.48, -1.20)	0.072	122	-18.64	**<0.0001**
		Ratio 1 (1:1)	6.95 (9.15, 17.52)	2.11	122	3.29	**0.0013**
		Ratio 2 (2:1)	13.33 (9.15, 17.52)	2.11	122	6.31	**<0.0001**
		N = 126; groups: Replication, 3; multiple R-squared: 0.76; adjusted R-squared: 0.75					
T. confusum	1	(Intercept)	11.69 (10.22, 13.15)	0.74	122	15.81	**<0.0001**
		Relative Arrival Times	0.41 (0.34, 0.48)	0.036	122	11.52	**<0.0001**
		Ratio 1 (1:1)	-2.17 (-4.24, -0.096)	1.05	122	-2.07	**0.0404**
		Ratio 2 (2:1)	-6.62 (-8.69, -4.55)	1.05	122	-6.33	**<0.0001**
		N = 126; groups: Replication, 3; multiple R-squared: 0.59; adjusted R-squared: 0.58					
	2	(Intercept)	25.40 (21.69, 29.12)	1.88	122	13.53	**<0.0001**
		Relative Arrival Times	1.21 (1.03, 1.38)	0.090	122	13.34	**<0.0001**
		Ratio 1 (1:1)	-6.31 (-11.57, -1.05)	2.66	122	-2.38	**0.019**
		Ratio 2 (2:1)	-13.88 (-19.14, -8.62)	2.66	122	-5.23	**<0.0001**
		N = 126; groups: Replication, 3; multiple R-squared: 0.63; adjusted R-squared: 0.62					
	3	(Intercept)	25.40 (21.74, 29.07)	1.85	122	13.72	**<0.0001**
		Relative Arrival Times	1.35 (1.17, 1.52)	0.089	122	15.12	**<0.0001**
		Ratio 1 (1:1)	-3.19 (-8.38, 2.00)	2.62	122	-1.22	0.23
		Ratio 2 (2:1)	-9.07 (-14.26, -3.89)	2.62	122	-3.46	**0.00074**
		N = 126; groups: Replication, 3; multiple R-squared: 0.66; adjusted R-squared: 0.66					

Table S5. Final densities of two-species vials. Negative relative arrival times means that *T. castaneum* arrives early.

**Relative Arrival Time**	**Number of Generation**	**Initial Egg Ratio (*T*:*F*)**	**Total Number of Eggs**	**Average Final Population, *T. castaneum***	**Standard Error, *T. castaneum* (n=3)**	**Average Final Population, *T. confusum***	**Standard Error, *T. confusum* (n=3)**
0	1	0.5	30	6.33333333	0.66666667	10.3333333	2.18581284
0	1	1	30	9.66666667	0.8819171	7.33333333	1.20185043
0	1	2	30	12.6666667	0.8819171	5.33333333	0.66666667
12	1	0.5	30	3	0.57735027	13.3333333	1.45296631
12	1	1	30	5.66666667	2.02758751	9.33333333	1.20185043
12	1	2	30	10.6666667	1.20185043	5.33333333	0.8819171
18	1	0.5	30	3	1.52752523	12	2.51661148
18	1	1	30	5.33333333	1.20185043	8	1.15470054
18	1	2	30	6.66666667	2.02758751	6.33333333	1.85592145
6	1	0.5	30	5	0.57735027	11	1
6	1	1	30	7.33333333	1.33333333	10	2.081666
6	1	2	30	10.3333333	1.85592145	4.66666667	1.33333333
-12	1	0.5	30	5.66666667	0.66666667	7	1.52752523
-12	1	1	30	7.66666667	0.8819171	4	1.15470054
-12	1	2	30	13.3333333	1.85592145	2.33333333	0.33333333
-18	1	0.5	30	6	1.73205081	1	0
-18	1	1	30	10	0.57735027	0	0
-18	1	2	30	11.3333333	2.02758751	1	0.57735027
-6	1	0.5	30	5.33333333	0.8819171	4.33333333	2.02758751
-6	1	1	30	6.66666667	0.8819171	8	1
-6	1	2	30	11.6666667	0.8819171	1.66666667	0.33333333
0	1	0.5	60	12	1.15470054	17.3333333	1.85592145
0	1	1	60	17.6666667	1.85592145	12	2.51661148
0	1	2	60	23.3333333	0.33333333	4.66666667	0.66666667
12	1	0.5	60	4	0.57735027	22.3333333	1.20185043
12	1	1	60	9.33333333	0.66666667	20	1.15470054
12	1	2	60	15.6666667	1.33333333	13	0.57735027
18	1	0.5	60	0.66666667	0.33333333	25.3333333	2.18581284
18	1	1	60	3.66666667	1.66666667	22	0.57735027
18	1	2	60	10.3333333	0.8819171	10.6666667	0.66666667
6	1	0.5	60	9.66666667	0.66666667	24.6666667	1.76383421
6	1	1	60	11.3333333	1.66666667	19	1.52752523
6	1	2	60	16	1	11.3333333	1.66666667

Table S5 (continued)

**Relative Arrival Time**	**Number of Generation**	**Initial Egg Ratio (*T*:*F*)**	**Total Number of Eggs**	**Average Final Population, *T. castaneum***	**Standard Error, *T. castaneum* (n=3)**	**Average Final Population, *T. confusum***	**Standard Error, *T. confusum* (n=3)**
-12	1	0.5	60	12.6666667	2.96273147	0.66666667	0.33333333
-12	1	1	60	17.3333333	0.8819171	2	2
-12	1	2	60	24.6666667	2.02758751	0.66666667	0.33333333
-18	1	0.5	60	9.33333333	0.8819171	1.66666667	0.33333333
-18	1	1	60	19	0.57735027	0	0
-18	1	2	60	22.3333333	1.76383421	0.33333333	0.33333333
-6	1	0.5	60	13.3333333	0.8819171	12.6666667	2.33333333
-6	1	1	60	19	1.52752523	11.6666667	3.17979734
-6	1	2	60	23.6666667	1.45296631	3.66666667	0.66666667
0	2	0.5	30	14.3333333	1.85592145	19.6666667	6.06446847
0	2	1	30	19	4	18.3333333	6.00925213
0	2	2	30	33.3333333	4.37162568	4	1.52752523
12	2	0.5	30	7.66666667	1.45296631	49	5.29150262
12	2	1	30	9.66666667	3.17979734	55	11.8462371
12	2	2	30	10	1	50.6666667	4.48454135
18	2	0.5	30	4.33333333	0.8819171	65.3333333	5.36449231
18	2	1	30	6.66666667	0.8819171	52	12.3423391
18	2	2	30	10	1.52752523	25.3333333	13.3832399
6	2	0.5	30	5.33333333	1.85592145	61.6666667	4.91030662
6	2	1	30	17	1	29.6666667	10.0884973
6	2	2	30	28.3333333	3.38296386	15.3333333	1.85592145
-12	2	0.5	30	25.3333333	1.20185043	4.66666667	0.66666667
-12	2	1	30	32.3333333	0.66666667	2.66666667	0.8819171
-12	2	2	30	38.3333333	0.8819171	0.33333333	0.33333333
-18	2	0.5	30	24.6666667	3.17979734	4.33333333	2.40370085
-18	2	1	30	27.6666667	2.02758751	1	0.57735027
-18	2	2	30	35	3.7859389	0	0
-6	2	0.5	30	27	3	6.33333333	2.18581284
-6	2	1	30	28.3333333	0.66666667	5.33333333	1.20185043
-6	2	2	30	38.3333333	4.70224533	1.66666667	0.8819171
0	2	0.5	60	17.3333333	3.92994204	23.6666667	4.91030662
0	2	1	60	20.6666667	1.45296631	20	3
0	2	2	60	28	2.081666	8.33333333	1.20185043
12	2	0.5	60	5.66666667	1.76383421	39.6666667	4.09606858

Table S5 (continued)

**Relative Arrival Time**	**Number of Generation**	**Initial Egg Ratio (*T*:*F*)**	**Total Number of Eggs**	**Average Final Population, *T. castaneum***	**Standard Error, *T. castaneum* (n=3)**	**Average Final Population, *T. confusum***	**Standard Error, *T. confusum* (n=3)**
12	2	1	60	14	0.57735027	27	4.35889894
12	2	2	60	22.6666667	2.33333333	17	3.05505046
18	2	0.5	60	6	0	37.6666667	1.33333333
18	2	1	60	9.33333333	2.72845092	23.3333333	3.84418753
18	2	2	60	11.6666667	2.33333333	15.6666667	0.8819171
6	2	0.5	60	13	2	28	3.60555128
6	2	1	60	19	4.35889894	21	2
6	2	2	60	22.6666667	2.60341656	16.6666667	0.66666667
-12	2	0.5	60	34.3333333	2.84800125	0.66666667	0.33333333
-12	2	1	60	33	2.30940108	2	0.57735027
-12	2	2	60	33	1.52752523	1	0.57735027
-18	2	0.5	60	35	5.19615242	2.33333333	0.66666667
-18	2	1	60	37.3333333	5.45690185	0.33333333	0.33333333
-18	2	2	60	36.3333333	2.66666667	2.33333333	0.8819171
-6	2	0.5	60	24	4.50924975	12.6666667	4.05517502
-6	2	1	60	26.6666667	1.76383421	9.66666667	0.8819171
-6	2	2	60	37	2.30940108	3	2
0	3	0.5	30	14.6666667	4.84194635	29.3333333	9.35117343
0	3	1	30	24.3333333	0.8819171	25.6666667	4.09606858
0	3	2	30	39.3333333	2.84800125	6.33333333	2.02758751
12	3	0.5	30	15	5.6862407	23.6666667	12.706079
12	3	1	30	7.66666667	1.45296631	53.6666667	2.96273147
12	3	2	30	11.6666667	3.48010217	43	10.1488916
18	3	0.5	30	11.6666667	2.33333333	37.6666667	8.41295298
18	3	1	30	11	3.51188458	33.6666667	9.35117343
18	3	2	30	9.66666667	1.85592145	40.3333333	6.35959468
6	3	0.5	30	12.3333333	2.84800125	47.6666667	5.66666667
6	3	1	30	16	2.51661148	45	9.01849951
6	3	2	30	21.6666667	3.52766841	32.3333333	8.64741451
-12	3	0.5	30	33.6666667	1.66666667	7.33333333	3.48010217
-12	3	1	30	49	8.08290377	1	1
-12	3	2	30	49.6666667	7.44610263	0.66666667	0.33333333
-18	3	0.5	30	43.6666667	13.1951169	3.66666667	2.18581284
-18	3	1	30	45.6666667	2.96273147	3.66666667	1.76383421

Table S5 (continued)

Relative Arrival Time	Number of Generation	Initial Egg Ratio (*T*:*F*)	Total Number of Eggs	Average Final Population, *T. castaneum*	Standard Error, *T. castaneum* (n=3)	Average Final Population, *T. confusum*	Standard Error, *T. confusum* (n=3)
-18	3	2	30	52	5.03322296	0.33333333	0.33333333
-6	3	0.5	30	18.6666667	7.51295178	35	14
-6	3	1	30	51	4.93288286	2	1
-6	3	2	30	47	3.46410162	2.66666667	0.66666667
0	3	0.5	60	21	2	29.6666667	4.33333333
0	3	1	60	31.6666667	2.02758751	17	1.52752523
0	3	2	60	49.6666667	8.11035004	5	1.15470054
12	3	0.5	60	5	2.88675135	41	3.21455025
12	3	1	60	9.66666667	2.40370085	53	2.30940108
12	3	2	60	15.6666667	1.33333333	42.6666667	3.38296386
18	3	0.5	60	4.33333333	0.66666667	57.6666667	2.72845092
18	3	1	60	7	0.57735027	40.6666667	4.91030662
18	3	2	60	17.3333333	1.33333333	30.3333333	2.02758751
6	3	0.5	60	10	1.52752523	37.6666667	8.74325137
6	3	1	60	16.6666667	1.66666667	32.6666667	0.8819171
6	3	2	60	28.3333333	1.76383421	24.6666667	2.60341656
-12	3	0.5	60	39	5.03322296	3	1.52752523
-12	3	1	60	56	2.30940108	0	0
-12	3	2	60	58	1	0	0
-18	3	0.5	60	52.3333333	4.33333333	1.33333333	0.8819171
-18	3	1	60	55	2.081666	0.66666667	0.33333333
-18	3	2	60	59	4.50924975	0	0
-6	3	0.5	60	50.3333333	3.17979734	1	0.57735027
-6	3	1	60	48.3333333	3.84418753	2.33333333	1.20185043
-6	3	2	60	59.3333333	4.05517502	0.33333333	0.33333333

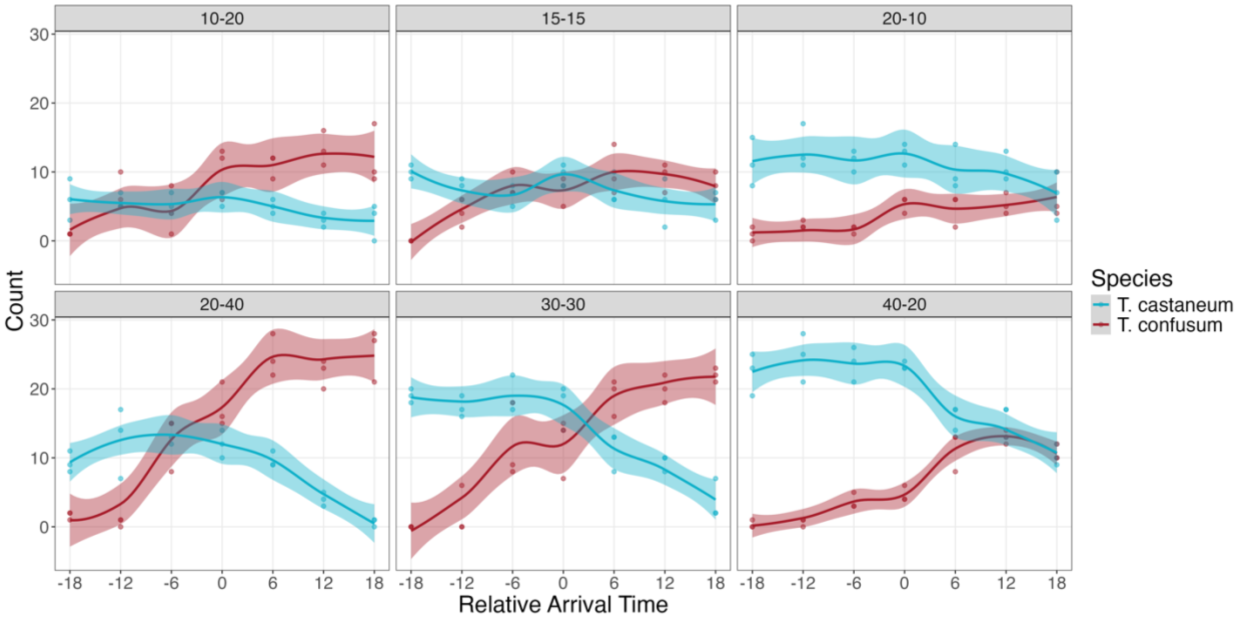

Figure S3. Final adult populations of vials with one generation. Numbers on top of each panel shows the initial population in the order of *T. castaneum* – *T. confusum*. Negative relative arrival times represents the early arrival of *T. castaneum*. The line and shades are means and standard errors smoothed by GAM for visualization purposes and do not represent any statistics performed in actual analyses.

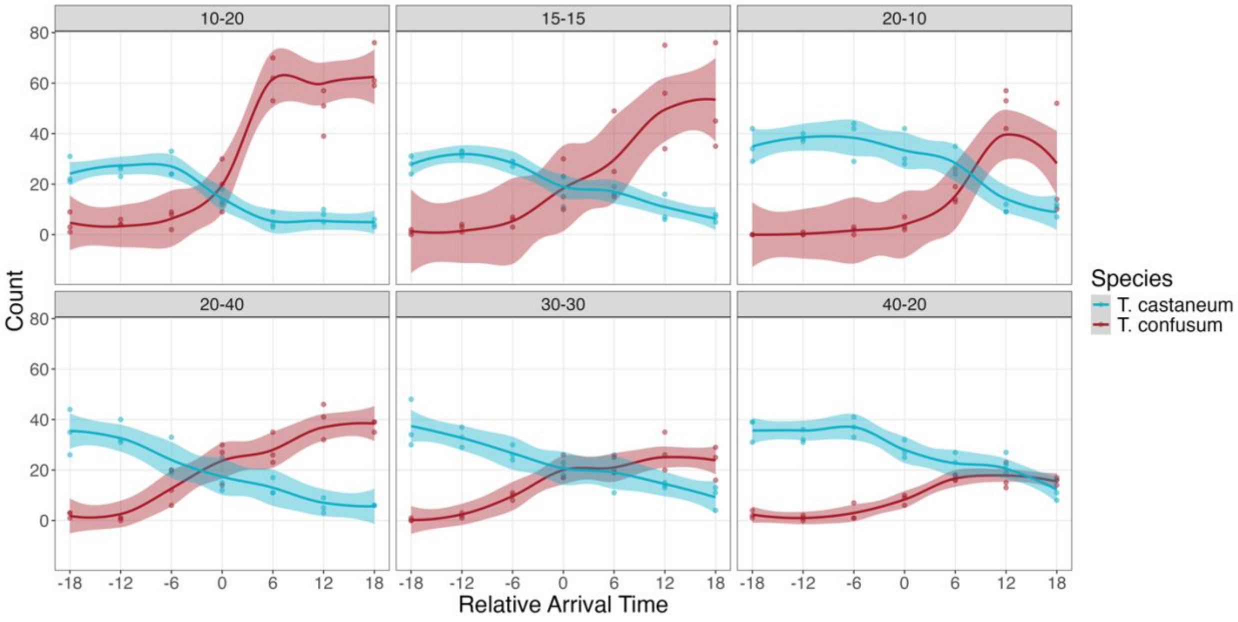

Figure S4. Final adult populations of vials with two generations. Numbers on top of each panel shows the initial population in the order of *T. castaneum* – *T. confusum*. Negative relative arrival times represents the early arrival of *T. castaneum*. The line and shades are means and standard errors smoothed by GAM for visualization purposes and do not represent any statistics performed in actual analyses.

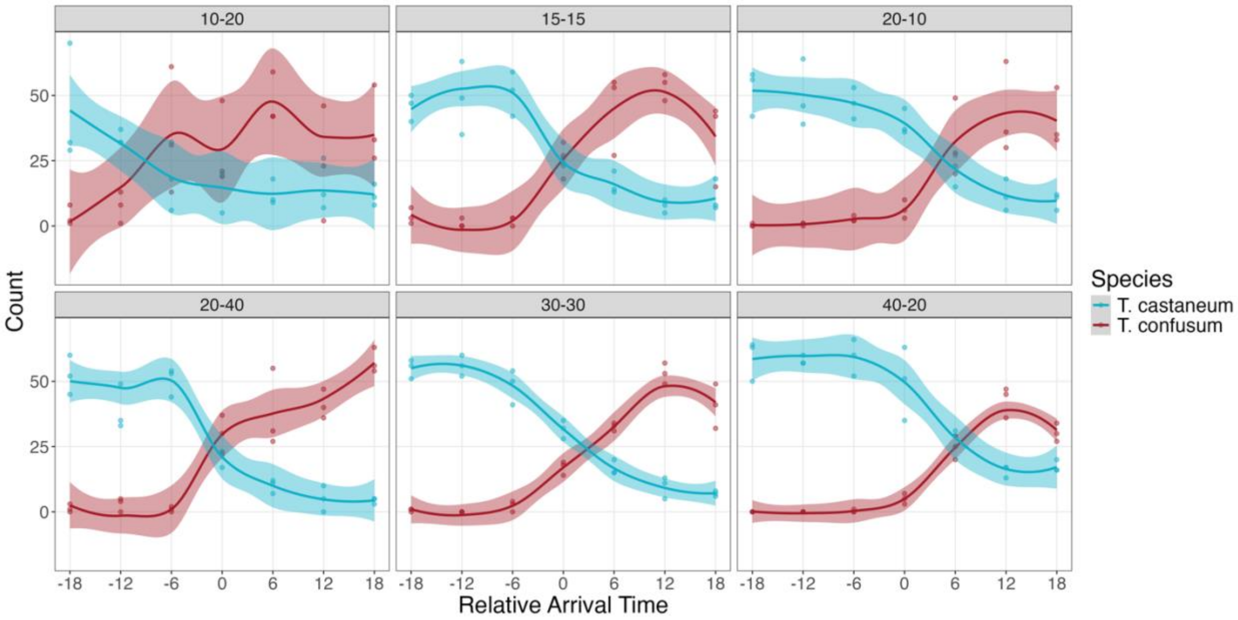

Figure S5. Final adult populations of vials with three generations. Numbers on top of each panel shows the initial population in the order of *T. castaneum* – *T. confusum*. Negative relative arrival times represents the early arrival of *T. castaneum*. The line and shades are means and standard errors smoothed by GAM for visualization purposes and do not represent any statistics performed in actual analyses.

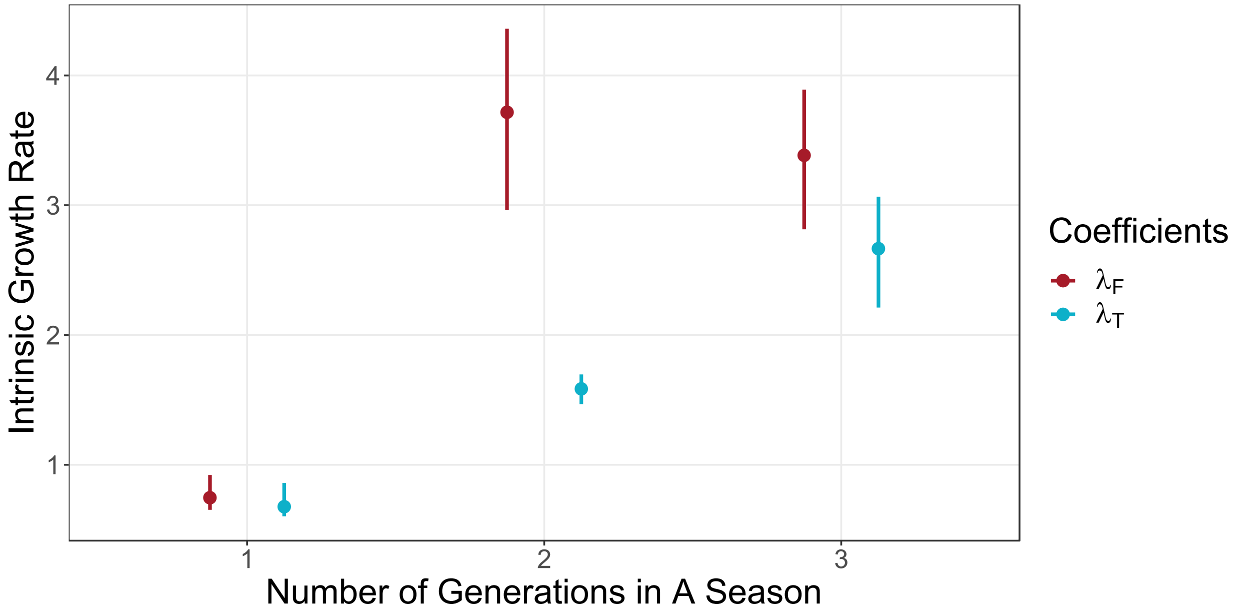

Figure S6. Fitted intrinsic growth rates from single-species vials. *F* represents *T. confusum*; *T* represents *T. castaneum*. Points show the medians, and line ranges show 80% credible intervals of the posterior distribution.

Section S3: Additional Experiments

Egg predation

To evaluate the potential of egg predation, we added 30 eggs of one species to one vial with 8 g medium containing 30 larvae of the other species at age 6, 12, and 18 days. Each vial is repeated six times. These conditions are identical to the starting conditions of the experimental vials. After one day, we sieved the vial to count number of remaining eggs. We used eggs collected less than 24 hours ago from the start of the experiment to minimize the possibility that any eggs hatched during the experiment. On average, larvae of *T. castaneum* consumed more eggs of *T. confusum* (one-sided Student's $t = -8.84$, df = 27.07, $p < 0.0001$, n = 18): the average egg survived in vials with *T. castaneum* is 11.50 (SE = 1.40), and the average number for *T. confusum* is 25.72 (SE = 0.80). For *T. castaneum*, the consumption increases with later stages (linear regression; slope of age is -0.60, $p = 0.031$, SE = 0.25, $t = -2.36$, n = 18).

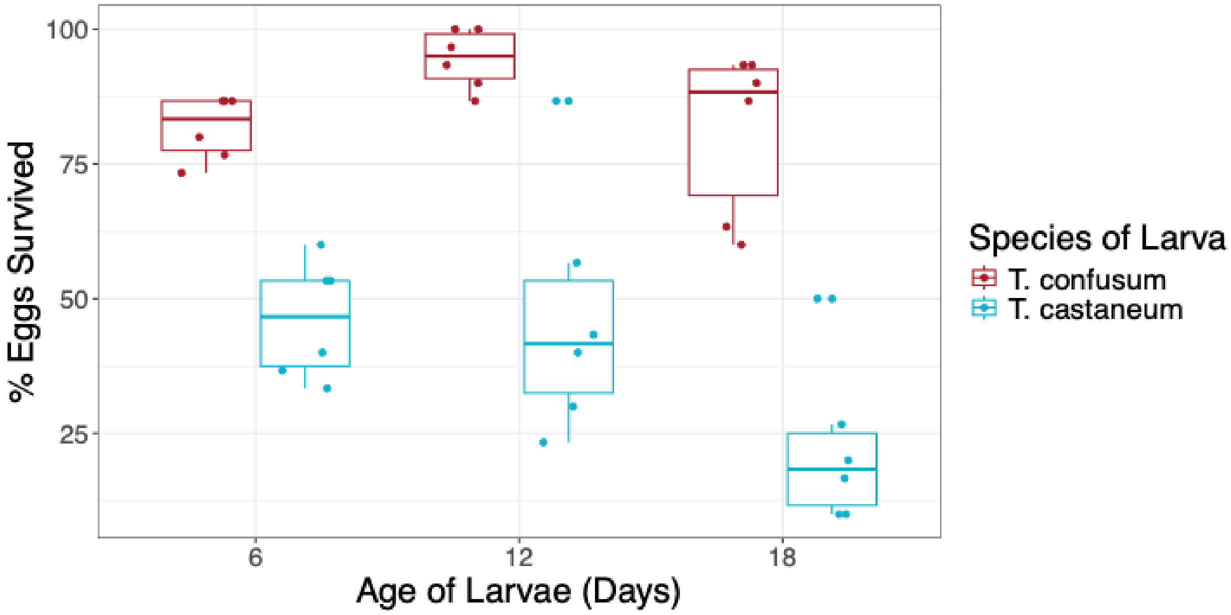

Figure S7. Predation of *T. castaneum* and *T. confusum* larvae on eggs of the other species.

Fecundity

We compared the fecundity of the two species by adding 30 randomly selected adults from our stock culture to 50 g medium. We counted the number of eggs daily for five consecutive days. *T. castaneum* produced less eggs than *T. confusum* (one-sided Student's $t = -9.07$, df = 4.72, $p = 0.00018$, n = 5), corresponding to our observations during maintenance of stock cultures and egg collection, that the latter can reach much higher abundance under lab conditions.

Table S6. Number of eggs laid by 30 randomly sampled individuals over five consecutive days.

**Species**	**Day 1**	**Day 2**	**Day 3**	**Day 4**	**Day 5**	**Average (SE)**
T. castaneum	72	102	107	103	99	96.60 (6.28)
T. confusum	291	371	266	297	249	294.80 (20.92)
